# Ubiquitination of the HPV Oncoprotein E6 Is Critical for E6/E6AP-Mediated p53 Degradation

**DOI:** 10.3389/fmicb.2019.02483

**Published:** 2019-10-31

**Authors:** Siying Li, Xiaoling Hong, Zhentong Wei, Min Xie, Wanying Li, Guanchen Liu, Haoran Guo, Jiaxin Yang, Wei Wei, Songling Zhang

**Affiliations:** ^1^Department of Obstetrics and Gynecology, The First Hospital of Jilin University, Changchun, China; ^2^Institute of Virology and AIDS Research, The First Hospital of Jilin University, Changchun, China

**Keywords:** HPV E6, p53, ubiquitination, R175H, USP15

## Abstract

High-risk Human papillomaviruses (HPVs) types are associated with more than 90% of premalignant and malignant squamous lesions of the uterine cervix. The E6 oncoprotein of high-risk HPVs is a key determinant in cell transformation because it induces the degradation of the host pro-apoptotic tumor suppressor p53. E6 recruits the intracellular ubiquitin ligase E6AP and subsequently induces proteasome-dependent p53 degradation. Neither E6 nor E6AP alone interact with p53; however, the precise mechanism of the functional regulation of the E6/E6AP/p53 complex is unclear. Here, we showed that the high-risk HPV E6 proteins are ubiquitinated during E6/E6AP/p53 complex assembly and degraded by the proteasome system. Increasing p53 expression enhanced E6/E6AP/p53 assembly and facilitated E6 ubiquitination and degradation. The dominant negative mutant of p53 R175H, which does not efficiently bind E6, decreased E6 ubiquitination and increased stability. Furthermore, we showed that the ubiquitin ligase E6AP is essential for E6 ubiquitination, and downregulation of E6AP expression increased E6 stability. We also showed that p53 R175H inhibited E6-mediated p53 degradation. Consistently, the host deubiquitinating enzyme USP15 removed ubiquitin chains from E6 proteins and inhibited E6-mediated p53 degradation. Crucially, ectopic expression of either p53 R175H or USP15 promoted p53-triggered apoptosis in human cervical cancer cells. Considering the importance of ubiquitinated E6 on p53 degradation, the disruption of E6 ubiquitination represents an attractive pharmacological intervention against HPV-positive human cervical cancer.

**Importance:**

Virtually 100% of cervical cancers are linked to HPV infection. Commercial HPV vaccines are estimated to prevent up to 90% of HPV-associated cancers, while they do not eliminate persistent HPV infections and have no effect on the progression to malignancy. Hence, the development of novel therapeutic interventions against HPV is urgently required. The HPV oncoprotein E6 binds to the intracellular E3 ubiquitin ligase E6AP and p53 resulting in the degradation of p53. In this study, we demonstrate that HPV E6 is ubiquitinated by E6AP in presence of p53. Crucially, ubiquitination of E6 is important for p53 degradation and blockage of E6 ubiquitination negatively interferes with E6-mediated p53 degradation and enhances the apoptotic effects of p53 and the cytotoxicity of DNA damage in HPV-positive cervical cancer cells. Importantly, our data suggest that the HPV oncogene E6 might be an optimal pharmacologic.

## Introduction

Human papillomaviruses (HPVs) are etiologically associated with several human cancers, especially human cervical cancer, the second leading cause of cancer-related death among women (de Martel et al., 2017). More than 120 HPV types have been characterized based on sequence homologies, but only a few have been found to be associated with cancer development. Among the high-risk HPV types, HPV16 and HPV18 account for approximately 70% of cervical cancers (de Sanjose et al., 2007; Moody and Laimins, 2010). HPV carries two oncogenes, *E6* and *E7*, which are overexpressed in HPV-positive cancers and have transforming and oncogenic properties in tissue culture and in animal models (Hoppe-Seyler et al., 2018). Mutational analyses have shown that the *E6* and *E7* genes of high-risk HPVs are sufficient for immortalization of human keratinocytes and fibroblasts (Hawley-Nelson et al., 1989; DeFilippis et al., 2003; de Sanjose et al., 2007). HPV16 and HPV18 E6 and E7 oncoproteins target the tumor suppressors p53 and retinoblastoma (pRB), respectively, for ubiquitin-mediated degradation, and induce cell proliferation, cell survival, genome instability, and innate immune evasion (Dyson et al., 1989; Scheffner et al., 1990, 1993; Huibregtse et al., 1991, 1993).

The E6 oncoproteins of high-risk HPVs, but not those of low-risk HPVs, interfere with the transcriptional activity of p53 and induce p53 degradation. E6-associated protein (E6AP), the founding member of the HECT E3 ubiquitin ligase family, has been found to mediate the binding between E6 and p53 (Scheffner et al., 1990, 1993; Huibregtse et al., 1991, 1993). The role of E6AP in E6-mediated p53 degradation has been well characterized *in vitro* and *in vivo*: E6, E6AP, and p53 form a complex that directs the ligase activity of E6AP to p53, subsequently triggering p53 degradation through the intracellular ubiquitin-proteasome system (Zanier et al., 2012; Vande Pol and Klingelhutz, 2013; Martinez-Zapien et al., 2016). While the major mechanism of E6-mediated oncogenesis is exerted through its ability to suppress the pro-apoptotic effects of p53, recent studies have demonstrated that E6 binds to several cellular proteins and induces their ubiquitination and degradation in an E6AP-dependent manner (White et al., 2012; Mesri et al., 2014).

The ubiquitin-proteasome system, a major clearance system for the balance of protein synthesis and degradation, ensures regulation of the protein pool in the cell (Isaacson and Ploegh, 2009). Protein ubiquitination is activated by a ubiquitin-activating enzyme (UAE, also called E1); the ubiquitin is then transferred to a ubiquitin-conjugating enzyme (E2). Finally, a ubiquitin ligase (E3) catalyzes the transfer of ubiquitin from E2 to the substrates. The ubiquitinated substrates are subsequently targeted to the 26S proteasome for degradation. The ubiquitin-proteasome system plays a central role in the cell under physiological and pathological conditions; in the case of HPVs, it is utilized to control the intracellular levels of the viral gene products at the post-translational level.

Deregulation of the ubiquitin-proteasome system contributes to the development of various diseases. In normal cells, the activity of E6AP is tightly controlled, and loss of E6AP expression causes the Angelman syndrome, a neurodevelopmental disorder (Tomaic and Banks, 2015), whereas increased E6AP expression has been associated with autism spectrum disorders (Samaco et al., 2005). Recent studies have revealed two inter-converting conformational states of E6AP, an active and a latent one (Mortensen et al., 2015). Crucially, HPV E6 acts as an allosteric activator of E6AP activity, increasing the accumulation of activated E6AP (Mortensen et al., 2015; Sailer et al., 2018). Yet, the mechanisms through which E6 regulates the assembly and function of the E6/E6AP/p53 complex are still not clear.

Human papillomavirus early genes are expressed at relatively low levels and have short half-live, while the late gene products L1 and L2 are much more stable. E6 proteins are short-lived and are stabilized in presence of proteasome inhibitors, suggesting the potential role of ubiquitination in the regulation of E6 stability and function (Stewart et al., 2004).

In present study, we found that HPV16 and HPV18 E6 proteins were ubiquitinated in an E6AP- and p53-dependent manner. Decreased E6AP or p53 expression increased E6 stability but impaired p53 degradation by E6. We also found that the p53 mutant R175H and the E6-binding partner USP15 stabilized E6 expression and inhibited E6-mediated p53 degradation by disrupting E6 ubiquitination. Importantly, overexpression of USP15 increased p53 expression levels and enhanced the cytotoxicity of etoposide-induced DNA damage in HPV-positive HeLa cells. Taken together, our data provide evidence for the importance of ubiquitination on HPV E6 activation and p53 degradation. Therefore, the interference with E6 ubiquitination might represent a new therapeutic approach for the treatment of HPV.

## Results

### Expression of p53 Facilitates the Proteasomal Degradation of HPV E6

**FIGURE 1:**
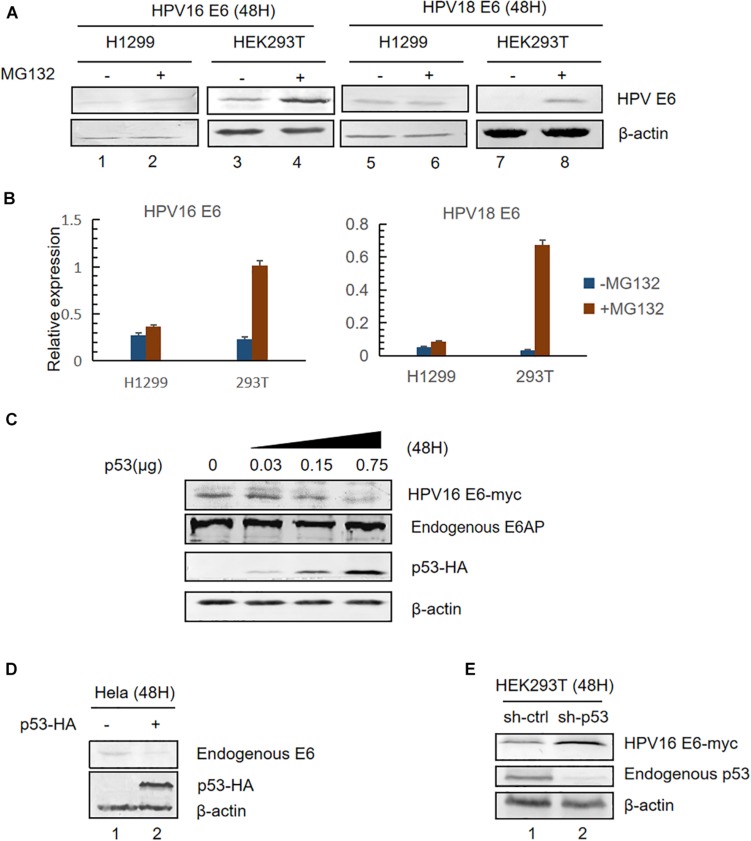
Effect of p53 expression levels on HPV E6. **(A)** HEK293T (p53-positive) or H1299 (p53-null) cells were transfected with the indicated plasmids. Thirty-six hours after transfection, the cells were treated with proteasome inhibitor (10 μM MG132) or DMSO and, 12 h later, were harvested for immunoblotting to detect E6 expression. **(B)** Relative expression of HPV16 E6 and HPV18 E6. Error bars represent the mean ± the standard deviation (SD) from triplicate experiments. **(C)** HEK293T cells were transfected with HPV16 E6 and dose-increased p53-expression plasmids. Forty-eight hours later, treated cells were collected and prepared for immunoblotting. **(D)** HeLa cells (HPV18-positive) were transfected with p53-expression plasmids, and endogenous E6 expression was detected by immunoblotting. **(E)** *p53*-silenced HEK293T cells were transfected with HPV16 E6-expressing plasmids and HPV16 E6 levels were detected by immunoblotting.

HPV E6 inactivates p53 by forming a stable bond with p53 and E6AP leading to ubiquitination-mediated degradation of p53 (Scheffner et al., 1990, 1993). Previous studies have shown that high-risk HPV E6 oncoproteins are short-lived proteins (Stewart et al., 2004). To investigate whether p53 levels affect E6 stability, we transfected plasmids overexpressing HPV16 or HPV18 E6 into HEK293T (p53-positive) or H1299 (p53-null) cells and, 36 h after transfection, the cells were treated with a proteasome inhibitor (10 μM MG132) or DMSO (control). Twelve hours later, the cells were harvested and E6 expression was detected by immunoblotting. Consistent with previous studies, we found that MG132 dramatically increased HPVs E6 expression in HEK293T cells (Figure 1A, lanes 3,4,7,8 and Figure 1B). However, there was no change in the levels of HPV E6 in H1299 cells (Figure 1A, lanes 1,2,5,6; Figure 1B).

Next, we transfected HEK293T with pHPV16 E6, overexpressing E6, and dose-increased p53 expression plasmids. Forty-eight hours later, treated cells were collected and subjected to immunoblotting. We noted that E6 levels remarkably decreased with the increasing p53 levels, but the expression of the E3 ubiquitin ligase E6AP is no significant differences (Figure 1C). Similar results were observed in HPV18-positive HeLa cells (Figure 1D). Furthermore, we silenced endogenous *p53* expression in HEK293T cells using shRNA. We found that HPV E6 expression increased in *p53*-silenced cells compared with control cells (Figure 1E). These data indicated that p53, target of E6, *trans*-regulates the stability of E6.

### Mutation of the p53-Binding Site Enhances HPV E6 Stability by Inhibiting Its Ubiquitination

**FIGURE 2:**
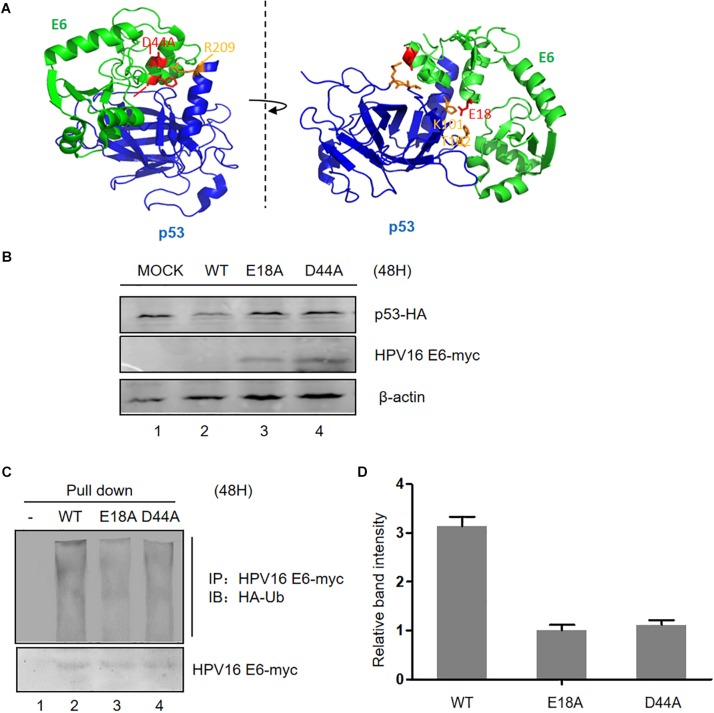
Effect of mutation of p53-binding sites on the stability of HPV E6. **(A)** According to the information from the crystal structure of the HPV E6/E6AP/p53 complex, we constructed three p53-binding site mutants of HPV E6 at the amino acids E18 and D44, respectively. **(B)** HEK293T cells were transfected with plasmids expressing wild type or mutated HPV16 E6 and p53 as indicated, and the expression of p53 and HPV16 E6 (WT or mutants) were detected by immunoblotting. **(C)** Cells were transfected with the indicated expression plasmids; after 36 h they were treated with 10 μM MG132 and 12 h later, harvested and subjected to immunoprecipitation (IP) with anti-myc-conjugated agarose beads. Polyubiquitinated HPV E6 was then detected for immunoprecipitation with an antibody against HA. **(D)** Relative expression of polyubiquitinated HPV16 E6 (WT or mutants). Error bars represent the mean ± the standard deviation (SD) from triplicate experiments.

According to the information provided from the crystal structure of the HPV E6/E6AP/P53 complex (Zanier et al., 2013), we constructed three plasmids expressing HPV E6 mutated at two sites (E18 and D44, respectively) in the p53-binding sites (Figure 2A). These mutants failed to induce p53 degradation, in the meantime, the expression of the two E6 mutants was dramatically higher than that of wild type E6 (Figure 2B). To measure the ubiquitination of the E6 mutants, we performed co-immunoprecipitation assays. Our data demonstrated that the ubiquitination of the E6 mutants is distinctly decreased compared with that of wild type E6 (Figures 2C,D). These data further indicate that p53 promotes E6 ubiquitination and degradation.

### Silencing of *E6AP* Increases the Stability of HPV E6

**FIGURE 3:**
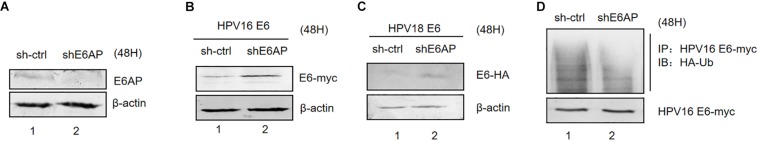
Effect of *E6AP* silencing on the stability of HPV E6. **(A)** *E6AP* knockdown was confirmed by immunoblotting. **(B)** HEK293T cells (E6AP-null) were transfected with HPV16 E6 or HPV18 E6. **(C)** After 48 h the cells were harvested, and protein levels analyzed by immunoblotting. β-actin acted as a control for transfection efficiency. **(D)** HEK293T cells (E6AP-null) were transfected with the indicated expression plasmids; after 36 h they were treated with 10 μM MG132 and 12 h later, harvested and subjected to immunoprecipitation (IP) with anti-myc-conjugated agarose beads. Polyubiquitinated HPV E6 was then detected for IP with an antibody against HA.

The E3 ubiquitin ligase E6AP is the crucial factor for HPV E6-induced p53 ubiquitination (Taylor and Stark, 2001). To address the roles of E6AP in E6 ubiquitination, we established silenced E6AP HEK293T cells using shRNAs. *E6AP* knockdown was confirmed by immunoblotting (Figure 3A). Downregulation of *E6AP* in HEK293T cells significantly increased HPV16 E6 and HPV18 E6 protein levels (Figures 3B,C). Furthermore, the ubiquitination of HPV16 E6 proteins was significantly decreased in the downreguation of *E6AP* in HEK293T cells (Figure 3D). Thus, during the E6/E6AP/p53 complex assembly, E6 is also ubiquitinated by E6AP.

### The p53 Dominant Negative Mutant R175H Is Resistant to E6-Mediated Degradation and Inhibits E6 Ubiquitination

**FIGURE 4:**
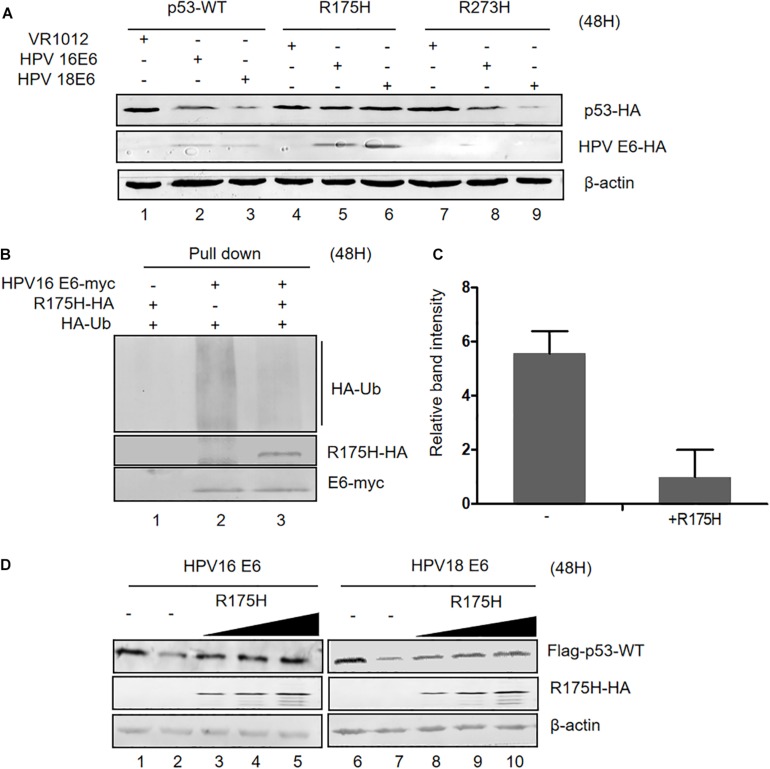
Effect of p53 R175H on E6-mediated degradation and E6 ubiquitination. **(A)** HEK293T cells were transfected with plasmids expressing wild type or mutated (R175H or R273H) p53 and HPV16 E6 or HPV18 E6 as indicated; 48 h later, cells were harvested and subjected to immunoblotting. **(B)** HEK293T cells were transfected with the indicated expression plasmids, and after 36 h they were treated with 10 μM MG132; 12 h later, cells were harvested and subjected to immunoprecipitation (IP) with anti-myc-conjugated agarose beads. Polyubiquitinated HPV16 E6 was then detected by immunoblotting with anti-HA antibody. **(C)** Relative expression of polyubiquitinated HPV16 E6. Error bars represent the mean ± the standard deviation (SD) from triplicate experiments. **(D)** HEK293T cells were transfected with plasmids expressing HPV16 E6 and dose-increased R175H expression plasmids; 48 h later, treated cells were collected and prepared for immunoblotting.

Somatic mutations of p53 are closely related with high risk of carcinogenesis. R175H and R273H are two characterized cancer-associated p53 mutations. We investigated the ability of HPV E6 to induce the degradation of these p53 mutants and found that, unlike wild type p53 and the R273H mutant, the R175H mutant was resistant to HPVs E6-mediated degradation (Figure 4A). Interestingly, we noticed that ectopic expression of p53 R175H remarkably increased E6 protein levels. Furthermore, in co-immunoprecipitation assays, we found that p53 R175H could be pulled down by HPV E6 (Figure 4B), indicating that this p53 mutant maintained the ability to interact with E6/E6AP. However, the ubiquitination of E6 proteins was significantly decreased in the presence of p53 R175H, which contributed to the stabilization of HPV E6 (Figures 4B,C).

Next, we investigated the effects of overexpression of p53 R175H on HPV E6 function. We transfected HEK293T cells with plasmids overexpressing HPV 16- or HPV 18-E6, p53 WT and increasing p53 R175H. Remarkably, ectopically expressed R175H conferred wild type p53 resistance to HPV16- and HPV18-E6-mediated degradation (Figure 4D). These results demonstrated that R175H acts as a dominant negative mutant inhibiting E6 ubiquitination and p53 degradation by E6.

### p53 R175H Restores the Pro-apoptotic Effects of p53 in HPV-Positive HeLa Cells

**FIGURE 5:**
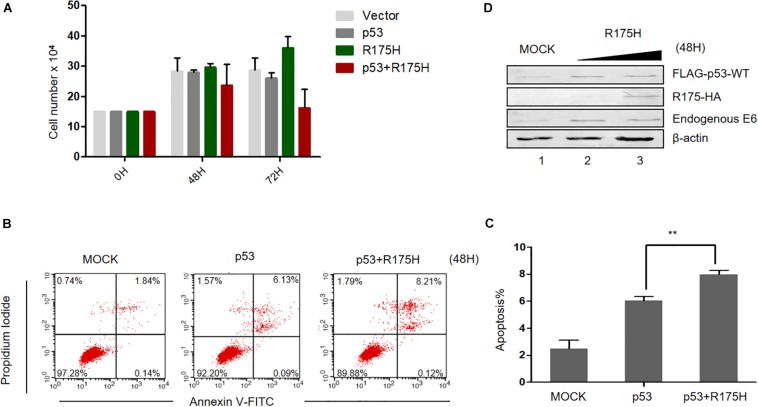
p53 R175H induces the apoptosis of HeLa cells by inhibiting the degradation of wild type p53 by HPV E6. **(A)** HeLa cells were transfected with the indicated expression plasmids; after 0, 48 or 72h, the cell number were counted by using hemocytometer. Error bars show the mean ± the standard deviation (SD). **(B)** Apoptosis of HeLa cells 48 h after transfection with the indicated expression plasmids, using Propidium Iodide (PI) and Annexin V-allophycocyanin labeling and flow cytometry analysis. **(C)** Apoptosis rates from the Annexin V-FITC/PI assays. Significant differences are denoted by ^∗∗^*P* < 0.001. **(D)** HeLa cells were transfected with the indicated plasmids and, 48 h later, they were harvested and subjected to immunoblotting.

Considering the effect of p53 R175H on HPV E6 activity, we then wondered whether this mutant could reestablish the tumor suppressor function of p53. We overexpressed p53 (WT or R175H)into HPV18-positive HeLa cells and measured the proliferation of the cells. We found that overexpression of p53-WT or R175H did not significantly affect the growth of HeLa cells, whereas the number of cells co-transfected with p53-WT and R175H dramatically decreased 72 h after transfection (Figure 5A). Furthermore, HeLa cells were co-transfected with plasmids encoding p53 (WT or R175H), and apoptosis was measured by flow cytometry. We found a modest increase of apoptosis in HeLa cells overexpressing wild type p53 (Figure 5B). However, the R175H mutant significantly enhanced the apoptotic effect of p53 (Figures 5B,C). Immunoblotting assays further showed that p53 R175H protected p53 from degradation by E6 (Figure 5D). These data revealed a close relationship between ubiquitination of E6 and E6-meidated p53 degradation.

### USP15 Enhances HPV E6 Protein Stability by Disrupting E6 Ubiquitination

**FIGURE 6:**
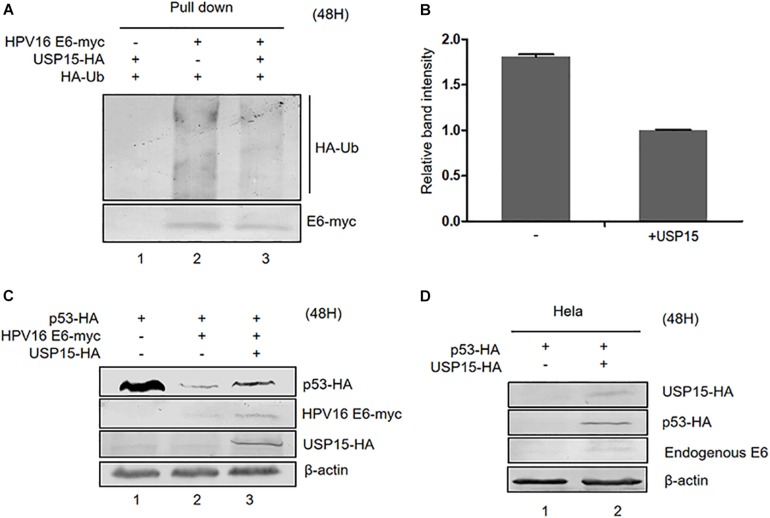
Effect of USP15 on E6-meditated p53 degradation and E6 ubiquitination. **(A)** HEK293T cells were transfected with the indicated expression plasmids; after 36 h they were treated with 10 μM MG132, and, 12 h later, cells were harvested and subjected to immunoprecipitation (IP) with anti-myc-conjugated agarose beads. Polyubiquitinated HPV16 E6 was then detected by immunoblotting with anti-HA antibody. **(B)** Relative expression of polyubiquitinated HPV16 E6. Error bars represent the mean ± the standard deviation (SD) from triplicate experiments. **(C)** HEK293T cells were transfected with indicated expression plasmids; 48 h later, cells were harvested and subjected to immunoblotting. **(D)** HeLa cells were transfected with indicated expression plasmids and, 48 h later, cells were harvested and subjected to immunoblotting.

In addition to E6AP, the deubiquitylating enzyme USP15 has been identified to be a HPV16 E6-associated protein. Previous studies have shown that USP15 increases HPV E6 stability, suggesting that ubiquitinated E6 might be a substrate for USP15 (Vos et al., 2009; Yaginuma et al., 2018). Co-immunoprecipitation assays in HPV16 E6-overexpressing HEK293T cells confirmed that overexpression of USP15 significantly decreased the levels of ubiquitinated E6 proteins (Figures 6A,B). Hence, we decided to investigate the role of USP15 in E6-mediated p53 degradation. We co-transfected HEK293T cells with p53-HA, pHPV16 E6 or pUSP15-HA and harvested the cells 48 h after transfection for immunoblotting assays. We found that overexpression of USP15 made p53 resistant to E6-induced degradation (Figure 6C). In addition, we also noticed that USP15 increased E6 protein levels, as previously described (Vos et al., 2009). Similarly, in HPV18-positive HeLa cells, overexpression of USP15 inhibited the degradation of p53 by HPV18 E6 (Figure 6D).

### USP15 Restores the Pro-apoptotic Effects of p53 in HPV-Positive HeLa Cells

**FIGURE 7:**
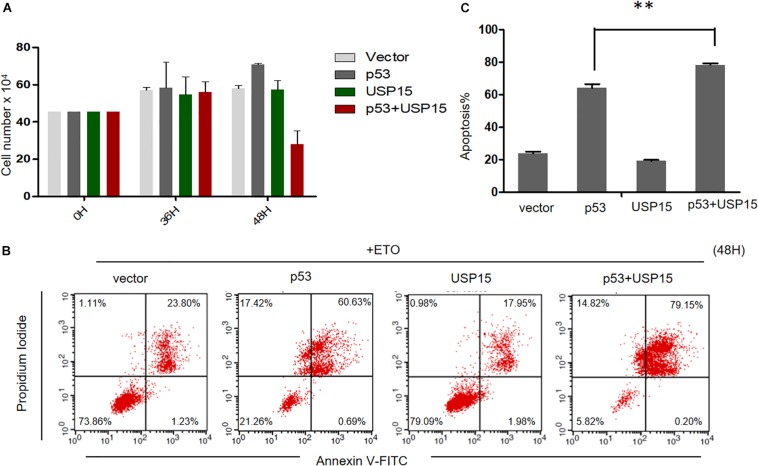
USP15 induces apoptosis and inhibits proliferation in HeLa cells. **(A)** HeLa cells were transfected with the indicated expression plasmids; after 0, 36 or 48h, the cell number were counted by using hemocytometer. Error bars show the mean ± the standard deviation (SD). **(B)** HeLa cells were transfected with the indicated expression plasmids; after 48 h, the cells were treated with 100 μM etoposide (Eto) for 12 h, followed by Propidium Iodide (PI) and Annexin V-allophycocyanin labeling and flow cytometry analysis. **(C)** Apoptosis rates from the Annexin V-FITC/PI assays. Significant differences are denoted by ^∗∗^*P* < 0.001.

Finally, we investigated the potential role of USP15 as a restriction factor for HPV E6-linked cancer cell survival. We overexpressed p53 or USP15 into HPV18-positive HeLa cells and measured the proliferation of the cells. We found that overexpression of p53 or USP15 did not significantly affect the growth of HeLa cells, whereas the number of cells co-transfected with p53 and USP15 dramatically decreased 48 h after transfection (Figure 7A). Furthermore, we demonstrated that co-expression of USP15 and p53, compared to overexpression of either USP15 or p53, significantly enhanced the cytotoxicity of the widely used chemotherapeutic agent etoposide (Eto), which induces DNA damage through inhibition of topoisomerase II (Figures 7B,C). These results demonstrate that the elevation of USP15 and p53 expression can repress the uncontrolled growth of HPV-positive cancer cells and trigger cell apoptosis.

## Discussion

Human papillomavirus-induced cancer is a health problem worldwide. Virtually 100% of cervical cancers are caused by HPV infection (de Martel et al., 2017). Cancer-causing HPV types carry two key oncogenes, E6 and E7, which are critical and necessary toward HPV-related disease progression and cancer. In HPV E6- and E7-transgenic mice, E6 primarily induces malignant tumors, whereas E7 induces benign tumors (Song et al., 2000). The major mechanism of E6 oncogenesis relies on E6 recruitment of E6AP, which, in turn, induces p53 degradation and suppresses cell apoptosis in infected cells. The investigation of the mechanisms of E6-mediated p53 degradation has important implications for understanding how cervical cancer develops and is maintained. In this study, we found that HPV E6 is ubiquitinated by E6AP in the presence of p53, and in this way it is targeted for degradation.

During E6-induced p53 degradation, HPV E6 proteins recognize the acidic leucine (L)–rich motifs containing the LxxLL consensus sequence within E6AP (Vande Pol and Klingelhutz, 2013; Zanier et al., 2013). Subsequently, the E6/E6AP heterodimer recognizes and degrades p53. However, the assembly of this complex is not a simple, bead-like connection model. The activity of the ubiquitin ligase E6AP is strictly limited in normal cells, and recent studies demonstrate that binding of HPV E6 induces conformational rearrangements in E6AP, promoting E6AP E3 ligase activity (Mortensen et al., 2015; Sailer et al., 2018). Here, we show that even in presence of E6AP, without p53 expression, E6 ubiquitination is blocked. This suggests that p53 bound to the complex triggers additional conformational changes in the E6/E6AP heterodimer and leads to ubiquitination of E6 by E6AP; this step is essential for E6-mediated p53 ubiquitination but also results in E6 degradation. Our studies have shown that the p53 dominant negative mutant R175H inhibits E6 ubiquitination and p53 degradation without interfering with the E6/E6AP/p53 complex assembly, further supporting our conclusions. Neither E6 nor E6AP are separately able to bind p53. In this regard, the assembly of the E6/E6AP/p53 complex is distinct from that mediated by other viral proteins (such as HIV Vif, Vpx etc.) recruited in ubiquitinating complexes. This unique assembly strategy may protect E6 from pre-degradation by E6AP in the absence of p53.

Accumulating evidence shows that E6 proteins are short-lived because of their ubiquitination, and the mechanisms of E6 stability are controversial (Kehmeier et al., 2002; Tomaic et al., 2009). Our data suggest that the ubiquitination of E6 is meditated by the ubiquitin ligase E6AP and enhanced by the target protein p53. Crucially, this ubiquitination is necessary for E6 to induce the ubiquitin ligase activity of E6AP to p53. We can hypothesize two possible models explaining this observation. Ubiquitinated E6 in the E6/E6AP/p53 complex might create a conformation that positions p53 close to E6AP; inhibition of E6 ubiquitination would interfere with the transfer of ubiquitin chains from E6AP to p53. Alternatively, E6 might be a viral ubiquitin ligase. Once the E6/E6AP/p53 complex is assembled, E6 proteins might be ubiquitinated by E6AP and transfer ubiquitin chains to p53. Notably, our findings have provided experimental evidence showing that E6 ubiquitination is druggable. Future studies should further investigate the mechanism through which E6 ubiquitination regulates E6-mediated p53 degradation.

The ubiquitin-specific peptidase USP15, a member of the largest subfamily of deubiquitinating enzymes (DUBs), binds to HPV E6 and regulates E6 protein stability (Vos et al., 2009; Yaginuma et al., 2018). Our data show that USP15 suppresses the ubiquitination of HPV E6, subsequently increasing the stability of HPV E6 (Figure 6). Importantly, we demonstrated that the effect of USP15 on E6 ubiquitination can efficiently block E6-mediated p53 turnover. Therefore, the ubiquitination of E6 is crucial for the E6/E6AP-mediated degradation of p53. Interestingly, the transcripts of *USP15* in HPV-positive cervix cancer tissues are significantly lower than in non-tumoral normal tissue (data not shown). In addition, a recent study has found that the interaction between USP15 and E6 contributes to viral immune evasion by suppressing RIG-I-mediated immune activation (Chiang et al., 2018), suggesting a complex relationship between the viral oncoprotein and host factors.

Human papillomavirus-induced cancers generally result from a persistent infection with high-risk HPV types in a process lasting several decades. Although HPV vaccines, based on the generation of virus-like particles through the HPV major structural protein L1 (Schiller and Muller, 2015), are available on the market, they are prophylactic with no influence on existing HPV infections and their progression to malignancy. Thus, HPV-linked carcinogenesis will remain a major health problem, and new treatment options are urgently required. Thus far, the specific therapeutic strategies targeting E6 are limited: research has focused on synthetic peptide ligands blocking E6/E6AP/p53 assembly (Dymalla et al., 2009), or depleting *E6* mRNA through RNAi or drugs (von Knebel Doeberitz et al., 1992; Goodwin and DiMaio, 2000; Hietanen et al., 2000; Jiang and Milner, 2002; Kochetkov et al., 2007). Inhibition of the ubiquitin ligase E6AP is an efficient way for restoring p53 functions, but E6AP activity is strictly controlled in normal cells and closely related to the development of several human diseases, therefore limiting its use as a drug target against HPV. In this study, we show that a p53 dominant negative mutant or USP15 increase E6 stability by blocking the ubiquitination of E6, impair E6-induced p53 degradation and enhance p53-dependent tumor suppression. These findings suggest that the disruption of E6 ubiquitination is a novel attractive target for therapeutic intervention on HPV-associated malignancies.

## Materials and Methods

### Plasmid Construction

The human wild type p53-HA, R175H-HA, R273H-HA and USP15-HA expression vectors were obtained from Addgene. FLAG-p53 was amplified using the primers 5′-AACTGCAGA CCATGGACTACAAGGACGACGATGACAAGATGGAGGAG CCGCAGTCAGAT -3′ (forward) and 5′-GAAGATCTTCAGT CTGAGTCAGGCCCTTC-3′ (reverse), to generate a product containing the *Pst*I and *Bgl*II sites and a C-terminal FLAG tag. The PCR product was cloned into the VR1012 vector to generate FLAG-p53. CDH-HAPV16E6, CDH-HAPV18E6 and CDH-MYC-PV16E6 were purchased from Addgene. HPV16 E6 E18A was generated with primers 5′-GTTACCACAGTTATGCACAGCGCTGCAAACAACTATACA TGATA-3′ (forward) and 5′-TATCATGTATAGTTGTTTGCAG CGCTGTGCATAACTGTGGTAAC-3′ (reverse); HPV16 E6 D44A was generated using the primers 5′-CTGCGAGTGAGGT ATATGCCTTTGCTTTTCGGGATTTATGC-3′ (forward) and 5′-GCATAAATCCCGAAAAGCAAAGGCATATACCTCACGT CGCAG-3′ (reverse); HPV16 E6 F47A was generated using primers 5′-TGAGGTATATGACTTTGCTCGTCGGGATT TATGCATAGTA-3′ (forward) and 5′-TACTATGCATAAATCCC GACGAGCAAAGTCATATACCTCA-3′ (reverse).

### Antibodies and Cell Culture

The following antibodies were used: rabbit monoclonal anti-HA (MAb, Covance, MMS-101R), rabbit polyclonal anti-E6AP/UBE3A (H00007337-M01, Novus Biologicals), mouse monoclonal anti-myc (Sigma, M5546), rabbit polyclonal anti-p53 (10442-1-AP, Proteintech) and mouse monoclonal anti-β-actin (Sigma, A3853).

HEK293T cells (AIDS Research Reagents Program) and the HPV 18-positive cell line HeLa (ATCC) were maintained in Dulbecco’s modified Eagle’s medium (DMEM) with 10% fetal bovine serum and penicillin/streptomycin. The non-small cell lung cancer cell line H1299 (ATCC) was cultured in RPMI 1640 medium with 10% FBS and penicillin/streptomycin. All cultured cell lines were maintained at 37°C in a humid atmosphere containing 5% CO_2_.

### Transfection, Immunoprecipitation and Immunoblotting

DNA transfection was carried out using Lipofectamine 2000 (Invitrogen) according to the manufacturer’s instructions. HEK293T cells were harvested 48 h after transfection, washed twice with cold PBS, and lysed in lysis buffer [150 mM Tris (pH 7.5), 150 mM NaCl, 1% Triton X-100, and complete protease inhibitor cocktail tablets (Roche)] at 4°C for 30 min, then centrifuged at 10,000 *g* for 30 min.

For Myc-tag immunoprecipitations, precleared cell lysates were mixed with anti-myc antibody-conjugated agarose beads (Santa Cruz Biotechnology) and incubated at 4°C for 3 h or overnight. Samples were then washed eight times with washing buffer [20 mM Tris (pH 7.5), 100 mM NaCl, 0.1 mM EDTA, and 0.05% Tween 20]. The immunoprecipitates were eluted with elution buffer (0.1 M Glycine–HCl, pH 2.0) and analyzed by SDS–PAGE and immunoblotting with the appropriate antibodies.

### shRNA-Mediated Knockdown and Retroviral Infection

The plasmids pRSV-Rev (12253), pMDLg/pRRE (12251), and pCMV-VSV-G (8454) were purchased from Addgene. *E6AP* knockdown was performed using a lentivector-based shRNA system (pLVX-shRNA Cloning and Lentivector Expression system, System Biosciences) according to the manufacturer’s recommendation. The *E6AP*-targeting shRNA primers were 5′-GATCCGCTAATAGAACGCTACTACCACCAGTTTCAAGAG AACTGGTGGTAGTAGCGTTCTATTAGTTTTTTACGCGTG- 3′ (forward) and 5′-AATTCACGCGTAAAAAACTAATAGAAC GCTACTACCACCAGTTCTCTTGAAACTGGTGGTAGTAGC GTTCTATTAGCG-3′ (reverse). The *p53*-targeting shRNA primers were 5′-GATCCGCGGCGCACAGAGGAAGAGAATC TCTTCAAGAGAGAGAATAATTTCCTGAATCTTGGCTTTT TTACGCGTG-3′ (forward) and 5′-AATTCACGCGTAAAAAA CGGCGCACAGAGGAAGAGAATCTCTCTCTTGAAGAGAA TAATTTCCTGAATCTTGGCCG-3′ (reverse). To generate VSV-G-pseudotyped shRNA virions, HEK293T cells were co-transfected with shRNAs, pCMV (coding for VSV-G), pRSV-Rev and pMDLg/pRRE. Two days after transfection, the cell culture medium was harvested. Cell debris was removed by centrifugation at 9838 *g* for 5 min and stored at −80°C. Virus input was adjusted using HEK293T as target cells. Virus infection of HEK293T cells treated with PMA (100 ng/mL for 2 days) was examined by western blot.

### Flow Cytometry

For the determination of cell death, cells were stained with propidium iodide and Annexin V-FITC (BD Pharmingen, San Diego, CA, United States) and analyzed on a flow cytometer (FACSCalibur, Becton Dickinson, Mount View, CA, United States). Electronic compensation of the instrument was done to exclude overlapping of the emission spectra. A total of 10,000 events were acquired for analysis using the CellQuest software (Becton Dickinson). Annexin V/propidium iodide-positive cells were regarded as apoptotic cells.

### Ubiquitination Assays

HEK293T cells transfected with plasmids overexpressing HA-Ub and the indicated proteins for 36 h were treated for 12 h with 10 μM MG132 (Sigma) to block proteasome degradation. Cells were then lysed in a buffer containing 2% SDS, 150 mM NaCl, 10 mM Tris–HCl and 1 mM DTT. The cell lysates were boiled immediately for 10 min to inactivate cellular ubiquitin hydrolases and preserve ubiquitin-protein conjugates. The heated lysates were then cooled and used for immunoprecipitation with an antibody against HA.

### Cell Counting

HeLa cells were cultured in 24-well plates under standard conditions until they reached 70% confluency. After DNA transfection, cell cultures were trypsinized with 0.25% Trypsin/0.1% EDTA for 3 min and then the cell suspensions were diluted with 0.4% trypan blue solution. Cells were counted using a hemocytometer.

### Statistical Analysis

All data were analyzed using the GraphPad Prism program (GraphPad Software Inc., San Diego, CA, United States). Statistical analysis of significance was performed using one-way ANOVA with LSD *Post hoc* multiple comparisons on raw data reads using the SPSS software (SPSS Inc., Chicago, IL, United States). *P* < 0.05 indicated significance.

## Data Availability Statement

The datasets generated for this study are available on request to the corresponding author.

## Author Contributions

SL, XH, MX, and WL performed the experiments. SL, GL, ZW, and JY analyzed the data. SL, WW, and HG wrote the manuscript with help from all authors. WW and SZ directed the project.

## Conflict of Interest

The authors declare that the research was conducted in the absence of any commercial or financial relationships that could be construed as a potential conflict of interest.
